# Modeling of Masked Droplet Deposition for Site-Controlled Ga Droplets

**DOI:** 10.3390/nano13030466

**Published:** 2023-01-23

**Authors:** Stefan Feddersen, Viktoryia Zolatanosha, Ahmed Alshaikh, Dirk Reuter, Christian Heyn

**Affiliations:** 1 Center for Hybrid Nanostructures (CHyN), University of Hamburg, Luruper Chaussee 149, D-22761 Hamburg, Germany; 2 Department of Physics, Paderborn University, Warburger Str. 100, D-33098 Paderborn, Germany

**Keywords:** quantum dots, site control, masked deposition, area-selective deposition, droplet density, droplet epitaxy, nucleation, Monte Carlo simulation, molecular beam epitaxy

## Abstract

Site-controlled Ga droplets on AlGaAs substrates are fabricated using area-selective deposition of Ga through apertures in a mask during molecular beam epitaxy (MBE). The Ga droplets can be crystallized into GaAs quantum dots using a crystallization step under As flux. In order to model the complex process, including the masked deposition of the droplets and a reduction of their number during a thermal annealing step, a multiscale kinetic Monte Carlo (mkMC) simulation of self-assembled Ga droplet formation on AlGaAs is expanded for area-selective deposition. The simulation has only two free model parameters: the activation energy for surface diffusion and the activation energy for thermal escape of adatoms from a droplet. Simulated droplet numbers within the opening of the aperture agree quantitatively with the experimental results down to the perfect site-control, with one droplet per aperture. However, the model parameters are different compared to those of the self-assembled droplet growth. We attribute this to the presence of the mask in close proximity to the surface, which modifies the local process temperature and the As background. This approach also explains the dependence of the model parameters on the size of the aperture.

## 1. Introduction

Secure information and communication technologies can greatly benefit from quantum effects wherein semiconductor quantum dots (QDs) represent essential building blocks as deterministic sources for single photons and entangled photon pairs [1,2,3]. However, the often-used self-assembled epitaxial QDs [4] are usually located on a semiconductor surface without significant lateral ordering. On the other side, the site-controlled generation of QDs on a surface is essential for the realization of advanced optical devices like quantum photonic integrated circuits [5]. Several approaches have been demonstrated for site-control QDs, often based on molecular beam epitaxy (MBE) technology [6]. We note that a true site control of the QD position requires more technical effort compared to a regular lateral ordering, which can be achieved by optimized self organization strategies. Two general concepts are applicable for MBE-based site control on a surface. First, modifications of the substrate surface before MBE growth cause a rearrangement of the planarly deposited material for site control [7,8,9,10,11,12,13,14,15,16,17,18,19,20]. Second, the material beam fluxes to the substrate surface are modified for a area-selective deposition [21,22,23]. We consider the second approach to be more flexible, since a patterned substrate often only allows geometries where the distances are close to the natural diffusion length of the unpatterned surface. On the other hand, a modulation of the beam fluxes e.g., by shadow masks, requires additional equipment which must be compatible with the restrictive requirements of the MBE method regarding the ultra-high purity of the used environment.

**Figure 1:**
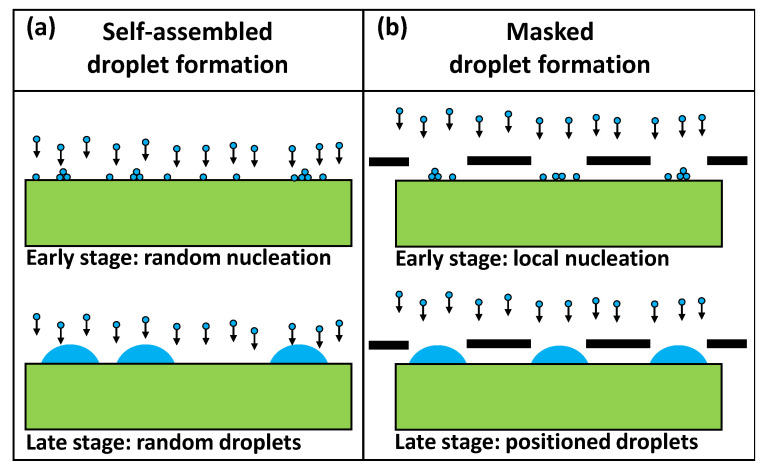
(**a**) Schematic of self-assembled droplet formation with planar deposition of the droplet material on a substrate, random nucleation, and formation of metal droplets without lateral order. (**b**) Schematic of an area-selective deposition process where Ga droplets are site controlled on an Al${}_{0.3}$Ga${}_{0.7}$As surface by apertures in a mask which consists of a 100 nm thick Si${}_{3}$N${}_{4}$-membrane on a Si(100) support wafer.

The present manuscript discusses the generation of site-controlled Ga droplets by area-selective deposition through apertures in a mask. Here, a shadow mask restricts the arrival of new material to areas below the apertures. Figure 1 illustrates the difference between self-assembled formation of droplets without lateral order and area-selective deposition through apertures in a mask for site-controlled droplets. The design of the mask is described in ref. [24] and the process in ref. [23]. For QD generation, the Ga droplets can be crystallized into GaAs quantum dots using a crystallization step under As flux (droplet epitaxy [25]). The central topic of this manuscript is a multiscale kinetic Monte Carlo (mkMC) simulation of the area-selective formation of Ga droplets by deposition through an aperture. The simulated surface morphologies are compared with experimental results for a parameterization of the model and for a better understanding of the complex mechanism behind the area-selective deposition and the subsequent thermal annealing step.

## 2. Masked Droplet Deposition

The present experimental approach for the generation of site-controlled GaAs QDs by area-selective droplet deposition is described in earlier publications [23,24]. Therefore, we give here only a brief summary of the major points.

The samples were fabricated in a multi-chamber solid-source MBE system using semi-insulating (100) GaAs wafers as substrates [23]. In a first step, MBE growth was performed without a mask, and a 100 nm thick Al${}_{0.3}$Ga${}_{0.7}$As layer was deposited at usual MBE process parameters. Then, the As flux was minimized by closing the As shutter and valve, the substrate temperature was reduced to 350 °C, and a Ga pre-coverage of 1.2 monolayers (ML) was deposited at a rate of 0.7 ML/s to create a Ga-terminated surface. After that, the substrate was cooled down to 100 °C and transferred into the preparation chamber. There, a mask–substrate sandwich was created under ultrahigh vacuum (UHV) conditions employing a special manipulator in the preparation chamber [23]. The mask is a nano-patterned 100 nm thick Si${}_{3}$N${}_{4}$-membrane on a Si(100) support wafer [24]. Electron beam lithography and reactive ion etching produced circular holes in the membrane of the mask with varied diameters *d* from 140 nm to 5 µm. We note that the masks are fully compatible with the demanding ultra-high vacuum requirements of the MBE technology. The mask is installed in close contact with the substrate surface. However, we expect that there is still a small gap between mask and substrate (probably <1 µm). After transferring the mask–substrate sandwich back to the growth chamber, different amounts of Ga were deposited now through the mask for area-selective deposition at 100 °C and minimized As flux. Then, after a 60 s pause, the mask–substrate sandwich was transferred into the preparation chamber. After mask removal, the substrate was transferred back to the growth chamber and annealed there for 5 min at a temperature of 400 °C and at minimized As flux.

## 3. Multiscale Kinetic Monte Carlo Simulation

The simulation model for the masked droplet deposition expands a previous model for the self-assembled formation of Al and Ga droplets on a GaAs surface [26]. Reference [26] compares two approaches to model droplet formation; one uses mean-field rate equations and the other a multiscale kinetic Monte Carlo (mkMC) simulation. Obviously, a mean field model is not compatible with the present masked deposition, where the material flux to the surface is laterally modulated. Therefore, we expand the mkMC model to now include the effects of the mask.

The mkMC simulation reflects an atomistic picture and approximates the substrate surface by a square simulation field with $m=m_{x}\times m_{y}$ surface sites. On an (001) AlGaAs surface, the distance between two surface sites is $d_{s}=a/2^{0.5}≃$ 0.40 nm, with the lattice constant *a*. The size of the simulation field $m=5d/d_{s}$ is adjusted according to the respective diameter *d* of the aperture (see Section 4). In contrast to the model for self-assembled droplet formation, where cyclic boundary conditions are assumed [26], we assume here that atoms diffusing out of the simulation field will not come back and, thus, are deleted. Considered objects on the surface are mobile atoms (monomers) and hemispherically-shaped droplets composed of s>1 atoms. Possible processes are the arrival of atoms from the vapor beam on the surface, surface diffusion of mobile monomers by nearest-neighbor hopping, nucleation events by collisions between migrating monomers, attachment of mobile monomers to droplets, and escape of atoms from droplets. As an extension of the original mkMC model, in addition, the coalescence of droplets is considered due to higher coverage with droplet material.

In the following, the modeling of the respective processes is described in more detail. The flux of atoms from the vapor beam to the surface is site dependent, with *F* within the circular opening in the mask; otherwise, *F* = 0. An atom arriving on the surface can result either in the formation of a new monomer, a nucleation event by a direct hit to another monomer, or in droplet growth by a direct hit. Monomers hop to a nearest-neighbor surface site with rate $D=\nu \exp\left[-E_{S}/\left(k_{B}T\right)\right]$, where *T* is the temperature, $\nu =2k_{B}T/h$ is a vibrational frequency [27], $k_{B}$ Boltzmann’s constant, *h* Planck’s constant, and $E_{S}$ the activation energy for surface diffusion. This diffusion of monomers can cause a site-change, a nucleation event, or the attachment to a droplet. In the latter case, the increasing droplet volume can cause the coalescence of two droplets. Here, the touching droplets merge to form a new droplet. The new size *s* is the sum of the two merging droplets, and the new position is the center of mass between them. After each droplet coalescence, the appearance of further coalescence events due to increasing droplet volume is checked recursively. Finally, atoms escape from a droplet composed of *s* atoms with rate $R_{E,s}=2\pi r\zeta \nu \exp\left[-E_{E}/\left(k_{B}T\right)\right]$, where $E_{E}$ is the activation energy for escape of monomers from droplets, $\zeta =\exp(r_{c}/r)$ describes the enhancement of the vapor pressure for small droplets due to the Gibbs-Thomson effect, $r=\sqrt[{3}]{3s/(2\pi )}$ is the droplet radius, $r_{c}=2\gamma V_{mol}/\left(N_{A}k_{B}T\right)$, γ is the surface tension (0.67 N/m for Ga), $V_{mol}$ the molar volume (11.8 $\times 10^{-6}$ m${}^{3}$/mol for Ga), and $N_{A}$ the Avogadro constant. An escape event from a droplet yields a new monomer at a random angle and distance *r* + 2 from the droplet center.

In ref. [26], it is described that an individual monomer performs several orders of magnitude more diffusion events compared to arrival plus escape. In order to speed-up the simulation and reduce the number of simulation steps, we replace monomer diffusion via a large number of nearest-neighbor hops by fewer jumps over longer distances [26]. This approximation is established by DeVita et al. as a multiscale kinetic Monte Carlo algorithm [28]. In detail, all diffusion events within the time interval $\tau_{I}=1/\left(mF+\sum_{s}n_{s}R_{E,s}\right)$, up to the occurrence of the next arrival or escape, are summarized. The monomer surface diffusion length is $\lambda =\sqrt{\tau D}$ according to the Einstein relation, with the diffusion time τ. In the time interval $\tau_{I}$ the diffusing monomer travels a distance $d_{I}=\sqrt{\tau_{I}D}$. Or, in other words, the rate $R_{I}=1/\tau_{I}=mF+\sum_{s}n_{s}R_{E,s}$ for traveling a distance $d_{I}$ by diffusion is given by the time interval up to the next arrival or escape. If there are other objects at a distance smaller than $d_{I}$, the diffusion can result in either a displacement of the monomer, in nucleation by collision with another monomer, or in attachment to a droplet. The probability *p* for a collision with another object depends on the circular segment r/(πd) covered by the object, where *d* is the distance and *r* is the radius of the object. This gives the nucleation rate $R_{N,ij}=rD/\left(\pi d_{ij}^{3}\right)$ at which monomer *i* collides with a second monomer *j*, with the distance $d_{ij}$ between both. Accordingly, the rate of attachment to a droplet *k* is $R_{A,ik}=rD/\left(\pi d_{ik}^{3}\right)$. In the mkMC model, the various rates sum up to a total activity rate

(1) $$R_{tot}=mF+\sum_{s}n_{s}R_{E,s}+\sum_{i}\left(R_{I}+\sum_{j\neq i}R_{N,ij}+\sum_{k}R_{A,ik}\right)$$

The rate $R_{tot}$ is used for the random selection of the next process in the simulation and for the calculation of the time interval $dt=1/R_{tot}$ up to the next process.

## 4. Results and Discussion

**Figure 2:**
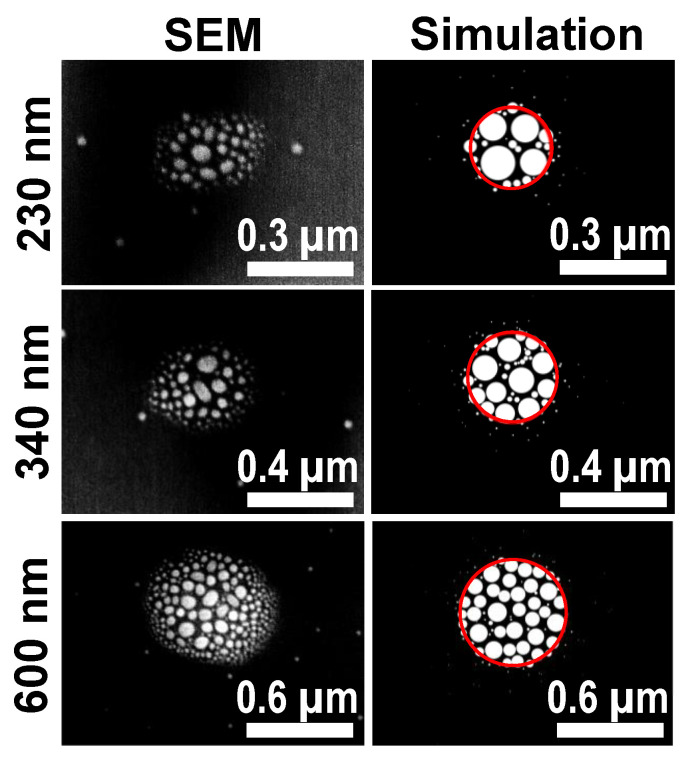
SEM images (**left**) of Ga droplets after area-selective deposition without annealing for varied diameters *d* of the mask aperture together with corresponding simulated surface morphologies (**right**). The red circles in the simulated surfaces indicate the size of the aperture. The experimental parameters are *F* = 0.35 ML/s, θ = 40 ML, and *T* = 100 °C. The simulations are performed using $E_{S}$ = 0.46 eV, $E_{E}$ = 1.24 eV, as well as an aperture size-dependent temperature of 50 °C at *d* = 230 nm, 65 °C at *d* = 340 nm, and 100 °C at *d* = 600 nm (see text).

For the area-selective deposition, an amount of θ = 40 ML of Ga is deposited through apertures in a mask at a flux of 0.35 ML/s and a low temperature, nominally of *T* = 100 °C (see Section 2). We note that measurements of such low temperatures are not very precise. Apertures of varied diameter *d* = 140 nm, 230 nm, 340 nm, and 600 nm are used. The following thermal annealing step of 5 min is performed at *T* = 400 °C. The average number of Ga droplets in the area of a mask aperture is taken from scanning electron microscopy (SEM) images. Examples are shown in Figure 2 where typically, data from 20 apertures are used to determine the average droplet number. The data in Table 1 clearly demonstrate that small apertures and a thermal annealing step are essential to achieve perfect site control with one droplet per aperture.

The average droplet number is also obtained from mkMC simulated surface morphologies and compared with the experimental values. Here, the size of the simulation field $m_{x}=m_{y}=5d/d_{s}$ is adjusted according to the respective diameter *d* of the aperture, with the distance $d_{s}$ between two surface lattice sites. For the two activation energies in the mkMC model, we start with values obtained in previous simulations [26] for self-assembled Ga droplet nucleation without a mask. For T≤ 300 °C the values are $E_{S}$ = 0.115 eV for monomer diffusion and $E_{E}$ = 1.24 eV for the escape of atoms from droplets. For T> 300 °C, the value of $E_{E}$ = 1.24 eV + 0.06 (T[°C] − 300)/100 eV is temperature dependent, which indicates that here, additional processes that modify the binding energy of adatoms to the droplets become relevant.

### 4.1. Simulation of the Area-Selective Deposition

For the area-selective deposition step at θ = 40 ML and *T* = 100 °C, simulations using the parameters for self-assembled Ga droplet formation ($E_{S}$ = 0.115 eV, $E_{E}$ = 1.24 eV) yield only a poor reproduction of the experimental droplet numbers in Table 1. To explain this discrepancy, we assume that the presence of a mask at a very small distance to the surface and with only small apertures modifies the local process parameters in the experiments. This can be the substrate temperature, the As background, or a combination of both. Furthermore, the usage of a different MBE chamber in the present experiments can also modify the As background. A modification of the As background is expected to influence the activation energies $E_{S}$ and $E_{E}$ in the simulation. To evaluate the respective influence, we have performed numerous simulation runs.

**Figure 3:**
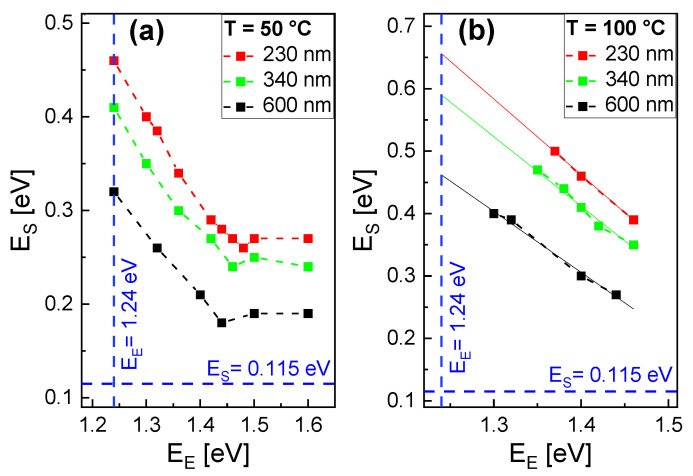
To parameterize the area-selective deposition step, pairs of $E_{E}$, $E_{S}$ are determined which yield simulated droplet numbers that are in agreement with the experimental values for (**a**) *T* = 50 °C and (**b**) *T* = 100 °C. Three aperture diameters *d* are considered as indicated. The continuous lines in (**b**) show results of linear fits. The dashed blue lines indicate the activation energies obtained for self-assembled droplet formation.

Figure 3 shows pairs of $E_{E}$, $E_{S}$ where the simulated droplet numbers agree with the experimental values after the deposition step. Two temperatures 50 °C and 100 °C as well as three aperture diameters *d* = 240 nm, 340 nm, and *d* = 600 nm are considered. As a general trend, a reduced $E_{S}$ is compensated by an increase in $E_{E}$. However, the data in Figure 3a indicate a saturation for $E_{E}$ above about 1.45 eV, where the escape of atoms from droplets becomes negligible small. Due to the saturation, there is no matching value of $E_{E}$ for the value of $E_{S}$ = 0.115 eV taken from the self-assembled droplet formation. For *T* = 100 °C, there are fewer simulated values, since the computation time increases exponentially with the temperature. Therefore, a linear regression is used to extrapolate for a smaller $E_{E}$.

To reduce the number of free model parameters, we assume in the following that only $E_{S}$ is sensitive to the mask, whereas $E_{E}$ = 1.24 eV agrees with the value of the self-assembled process. This approach reflects the known influence of an As background on the average activation energy for surface diffusion during GaAs epitaxy [29]. Here, a higher $E_{S}$ corresponds to a higher As background. In the present experiments, the mask can induce a higher As background and, thus, an increasing $E_{S}$ at a smaller *d*. The second column in Table 2 gives values of $E_{S}$ which provide agreement with the experiments as a function of *d* for constant $E_{E}$ = 1.24 eV and *T* = 100 °C.

**Figure 4:**
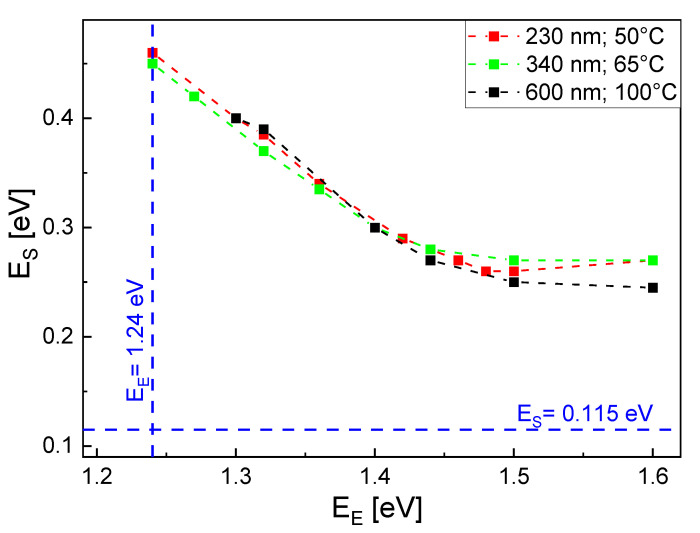
Pairs of $E_{E}$ and $E_{S}$ which yield simulated droplet numbers in agreement with the experimental values after deposition. The temperature is assumed to depend on the aperture diameter *d* as indicated. The dashed blue lines indicate the activation energies obtained for self-assembled droplet formation.

In a second approach to explaining the influence of the aperture size, the local temperature *T* can depend on *d*. Figure 4 shows pairs of $E_{E}$ and $E_{S}$ with agreement between simulation and experiment for adjusted values of *T*. Interestingly, again due to a saturation, the value of $E_{S}$ is always above that for the self-assembled process. This indicates an elevated As background even in the case of a *d*-dependent temperature. Assuming a constant $E_{E}$ = 1.24 eV and $E_{S}$ = 0.46 eV, the adjusted temperatures are given in the third column of Table 2. A comparison between experimental droplet morphologies and the simulation results obtained using this approach are shown in Figure 2.

To summarize this part, the area-selective deposition related results indicate that the activation energy $E_{S}$ for surface diffusion is higher compared to the self-assembled droplet formation. This is probably related to a higher As background which can be caused by the usage of a different MBE chamber or by the presence of the mask. The reason for the additional dependence of the droplet number on the aperture size is not unequivocal, and the effect can be explained by an aperture-size dependence either of the temperature or of the As background and, thus, of $E_{S}$ and possibly also of $E_{E}$.

### 4.2. Simulation of the Thermal Annealing Step

**Figure 5:**
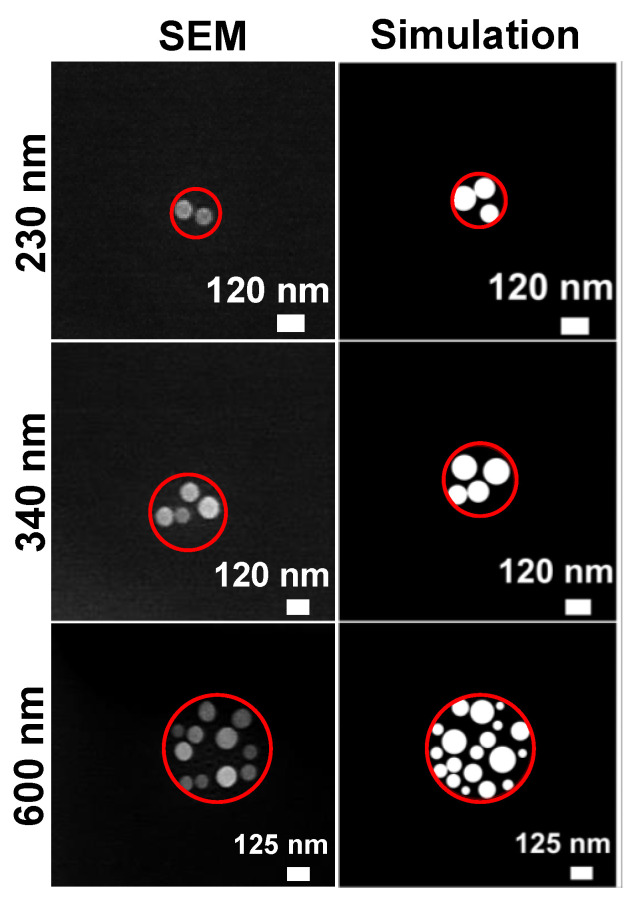
SEM images (**left**) of Ga droplets after area-selective deposition and thermal annealing for varied diameters *d* of the mask aperture, together with corresponding simulated surface morphologies (**right**). The red circles indicate the size of the aperture. The experimental and simulation parameters for the area-selective deposition step are as in Figure 2. Thermal annealing is performed for 5 min at *T* = 400 °C. The simulations of the annealing step use a *T*-dependent $E_{E}$, as is described in the text.

As a final process step, the samples are annealed without a mask for 5 min at *T* = 400 °C. This step reduces the droplet number substantially, as is visible in Table 1. Figure 5 shows examples of area-selective Ga droplets after annealing. Clearly visible is the strong influence of the aperture size on the droplet number.

The simulations of the annealing step start from the aperture-size dependent droplet morphologies which are described in Figure 2. Only the activation energy for the escape of atoms from droplets is modified at the high temperature, according to $E_{E}$ = 1.24 eV + 0.06 (T[°C] − 300)/100 eV (see Section 3). A comparison between experimental and simulated droplet morphologies in Figure 5 indicates that the model nicely reproduces the experimental trend. However, the simulation produces slightly higher droplet numbers. This deviation may be caused by the idealized model assumptions. As described in Section 3, the model considers an abrupt border between the area below the aperture with a constant impinging flux *F* and the area shadowed by the masked with *F* = 0. In the experiments, the geometry of the evaporation cell in combination with the distance between mask and substrate will lead to an additional transition region with a nonuniform flux and an enlarged diameter of the deposition area. Furthermore, the experiments indicate that a significant fraction of the deposited Ga is not found in the droplets (50% and more) [23]. This loss of the droplet material is attributed to an outdiffusion under the area shadowed by the mask. The simulations also indicate a material loss, but only up to 10%, which agrees with the higher droplet numbers in comparison to the experiments.

**Figure 6:**
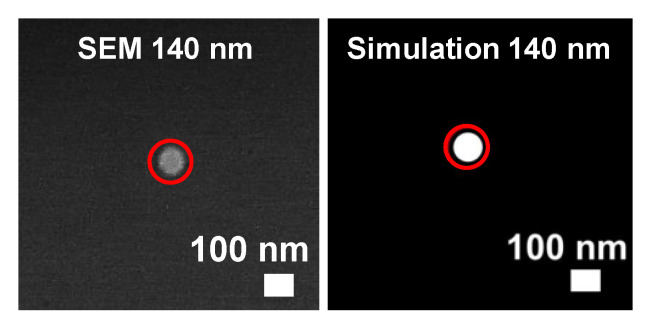
SEM image (**left**) of a single site-controlled Ga droplet for *d* = 140 nm together with a corresponding simulated surface morphology (**right**). The red circles indicate the size of the aperture. The experimental and simulation parameters are as in Figure 5. In the simulations, during deposition *T* = 50 °C is assumed (see Section 4.1).

For a demonstration of perfect site control with one droplet per aperture, a further sample was fabricated using a smaller aperture diameter of *d* = 140 nm. The other parameters are as in Figure 2 and Figure 5. Figure 6 demonstrates the creation of a single site-controlled Ga droplet using this small aperture. In the simulations, a temperature of *T* = 50 °C is assumed during deposition, which reflects the value for *d* = 230 nm (see Section 4.1). The diameter of the droplet taken from SEM is 100 nm, and the simulated droplet (Figure 6) has a very similar diameter of 98 nm.

**Figure 7:**
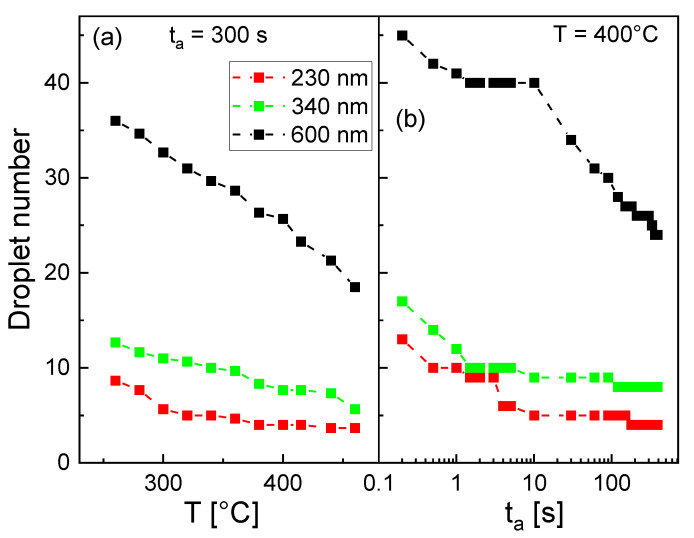
Simulated droplet number after annealing (**a**) as a function of annealing temperature *T* and (**b**) as a function of the annealing time $t_{a}$ for different aperture diameters *d*.

In order to study the annealing process in more detail, we have performed simulation runs with varied annealing temperatures *T* and annealing times $t_{a}$. Results are plotted in Figure 7. Clearly visible is the decreasing droplet number at a higher *T* and a longer annealing time. This reduction is attributed to two effects. First, the droplet coarsening by Qstwald ripening is well known to reduce the droplet number [30]. Second, the diffusion of material to areas below the mask far away from the aperture will also reduce the droplet number. We note that these results confirm the choice of the experimental annealing parameters *T* = 400 °C, $t_{a}$ = 300 s, and d≤ 230 nm as useful for the realization of perfect site control with one Ga droplet per aperture. Using such parameters, the fabrication of a perfectly ordered array of Ga droplets by deposition through a mask was demonstrated [23].

## 5. Conclusions

A multiscale kinetice Monte Carlo simulation [26] of self-assembled Ga or Al droplet formation on AlGaAs surfaces is expanded for area-selected deposition. Model results are compared with experimental data on the area-selective deposition [23] of Ga droplets through apertures in a mask [24]. In the experiments, a thermal annealing step after the area-selective deposition is very important for the reduction of droplet density down to one site-controlled droplet per aperture. The model is able to quantitatively reproduce the experimental behavior, including the area-selective deposition and the annealing step. However, the model parameters need to be changed in comparison to the self-assembled droplet growth. We assume here that the mask in close proximity to the surface modifies the local process temperature and the As background. A higher As background is expected to yield a higher activation energy for surface diffusion [29]. This approach also explains the dependence of the model parameters on the size of the aperture.

For a functionalization, the studied site-controlled Ga droplets can be crystallized into GaAs quantum dots under an arsenic flux. This method is established as droplet epitaxy (DE) [25,31]. We assume that the position and number of the Ga droplets will not change during crystallization, so that the site-control of the initial droplets is also maintained for the GaAs quantum dots. Droplet epitaxial quantum dots are promising building blocks for quantum photonic devices and quantum networks [25]. In an alternative approach, we expect that the masked deposition technique can be used also for the fabrication of site-controlled Al droplets. Al droplets allow the generation of various types of quantum structures through the local droplet etching (LDE) technique [32,33]. LDE takes place at much higher temperatures compared to DE, where the gradient of the As concentration causes a substantial diffusion of As from the AlGaAs substrate into the Al droplets. This yields the formation of nanoholes in the AlGaAs surface, which can be filled, e.g., with GaAs for the generation of quantum structures. Both concepts, DE and LDE, would be greatly improved by a site control of the resulting quantum structures. We expect that here, the present simulation model will be helpful for optimizing the process parameters.

## Figures and Tables

**Table 1:** Experimental average number of Ga droplets in the area of mask apertures of varied diameters *d*, determined using SEM.

*d* [nm]	Droplets after Deposition	Droplets after Annealing
230	20.8 ± 1.22	1.1 ± 0.07
340	34.3 ± 1.36	2.4 ± 0.21
600	52.0 ± 1.90	10.8 ± 0.41

**Table 2:** Two approaches for simulation parameters which reproduce the experimental droplet numbers after area-selective deposition for different aperture diameters *d*. $E_{E}$ = 1.24 eV is assumed to be constant.

*d* [nm]	$E_{S}$ [eV] at *T* = 100 °C	*T* [°C] at $E_{S}$ = 0.46 eV
230	0.66	50
340	0.59	65
600	0.46	100

## Data Availability

Not applicable.
